# Global Mapping of Transposon Location

**DOI:** 10.1371/journal.pgen.0020212

**Published:** 2006-12-15

**Authors:** Abram Gabriel, Johannes Dapprich, Mark Kunkel, David Gresham, Stephen C Pratt, Maitreya J Dunham

**Affiliations:** 1 Department of Molecular Biology and Biochemistry, Rutgers University, Piscataway, New Jersey, United States of America; 2 Generation Biotech, Lawrenceville, New Jersey, United States of America; 3 Lewis-Sigler Institute, Princeton University, Princeton, New Jersey, United States of America; 4 Department of Molecular Biology, Princeton University, Princeton, New Jersey, United States of America; 5 Department of Ecology and Evolutionary Biology, Princeton University, Princeton, New Jersey, United States of America; Fred Hutchinson Cancer Research Center, United States of America

## Abstract

Transposable genetic elements are ubiquitous, yet their presence or absence at any given position within a genome can vary between individual cells, tissues, or strains. Transposable elements have profound impacts on host genomes by altering gene expression, assisting in genomic rearrangements, causing insertional mutations, and serving as sources of phenotypic variation. Characterizing a genome's full complement of transposons requires whole genome sequencing, precluding simple studies of the impact of transposition on interindividual variation. Here, we describe a global mapping approach for identifying transposon locations in any genome, using a combination of transposon-specific DNA extraction and microarray-based comparative hybridization analysis. We use this approach to map the repertoire of endogenous transposons in different laboratory strains of Saccharomyces cerevisiae and demonstrate that transposons are a source of extensive genomic variation. We also apply this method to mapping bacterial transposon insertion sites in a yeast genomic library. This unique whole genome view of transposon location will facilitate our exploration of transposon dynamics, as well as defining bases for individual differences and adaptive potential.

## Synopsis

Transposons, or mobile DNA sequences—first described by Barbara McClintock—are interesting and important residents of all genomes. They are involved in gene creation and regulation, chromosome evolution, and generation of mutations, events that can occur on hugely varying time scales, from millions of years to mere days in the lab. Some transposons have even been “tamed” by geneticists for use as tools for marking genes and making mutations. In yeast, genome sequencing has given us a snapshot of transposons present in one strain at one particular time. The authors developed a method to easily, accurately, and globally track transposons in order to study how their locations change in different strains or during an experiment. The method involves finding pieces of DNA that contain the ends of transposons along with neighboring DNA and attaching these segments to magnetic beads. A magnet is then used to separate the selected DNAs away from the rest of the genome. The transposon-associated DNA is labeled with dyes and applied to a microarray, a glass slide with over 40,000 unique sequence features of yeast DNA attached. Each feature that lights up with the dye marks a transposon location. This new technique allows investigators to easily identify specific strains, to accurately monitor mobile portions of the genome, and to determine the role of transposons in phenotypic differences.

## Introduction

The genomes of all organisms studied have been populated, over evolutionary time, by different classes of transposable elements. These multicopy genetic elements, first postulated by Barbara McClintock, are regulated at many levels to suppress their movement: such movement has been shown to result in genetic diseases in humans [1], hybrid dysgenesis and sterility in *Drosophila* [2], spread of antibiotic resistance in bacteria [3], and, generally, insertional activation or inactivation of nearby genes [4,5]. Their effects on host genomes can be more widespread and subtle. The presence of an L1 retrotransposon in the intron of a gene can affect that gene's expression, by slowing of transcription through the L1 sequence [6]. Polymorphic transposon sequences within genes can result in allele-specific alternative splicing patterns with formation of new exons [7]. Mammalian L1 elements may be involved in neuronal somatic diversification [8]. In addition to events directly caused by polymorphic transposon insertions, the characteristic multicopy, dispersed organization of transposons throughout genomes results in their appearance at breakpoints of translocations, inversions, duplications, and deletions in tumor cells [9–11]. In *Saccharomyces cerevisiae,* transposon-associated chromosomal rearrangements may be selectively advantageous, in double strand break repair [12–16] and in experimental evolution studies [17–20]. Further, they may be involved in speciation and natural genome evolution [21–23]. Thus, differences in placement of transposons in individual genomes could cause or at least correlate with phenotypic differences.

The model eukaryote S. cerevisiae has been at the forefront of studies of retrotransposons, i.e., transposons that use reverse transcriptase for their replication and that copy and paste themselves to new genomic locations. Several distinct families of retrotransposons, or “Tys,” have been identified in this organism, both anecdotally and systematically through the genome sequencing effort. In the only fully sequenced S. cerevisiae strain, S288c, the most abundant transposons are Ty1 (31 copies) and Ty2 (13 copies). These closely related 5.9 kb full-length elements consist of two overlapping open reading frames (ORFs), each of which encode several proteins. The coding regions are flanked by nearly identical ∼330-bp long terminal repeats (LTRs). Ty4 (three copies) is a distinct and less abundant element with a similar structure. Ty3 (two copies) is another distinct element, with a different arrangement of protein-coding segments. Ty5 is a vestigial element, with no intact copies in the S. cerevisiae genome [3]. The insertion-site preferences of these different families are characteristic, with most Ty1 and Ty2, and all Ty3 and Ty4 elements found near tRNA sequences [24], and Ty5 fragments found within silenced DNA [25]. For each family of full-length Ty elements there are an order of magnitude more solo LTR elements dispersed through the genome. These are thought to arise by LTR–LTR recombination of full-length elements, with looping out of the internal regions.

The complete sequence of strain S288c provides a snapshot of retrotransposon positions in one S. cerevisiae strain at one point in time [26]. But transposons are dynamic, and cell-specific or strain-specific new insertions, recombinational losses, and potential rearrangements result in a much more complex and interesting picture than can be gleaned from a single complete genome sequence [27]. New endogenous transposon locations are typically discovered serendipitously or through large-scale whole genome sequencing projects. Even with whole genome sequencing, repetitive transposon sequences can greatly complicate assembly of contigs. Modified transposons, used as insertional mutagens and library markers, are identified through inverse PCR, vectorette PCR, thermal asymmetric interlaced PCR, or other imprecise, time-consuming, and potentially biased techniques.

We have developed a comparative approach to identify the location of transposons throughout a genome. Here, we describe in S. cerevisiae a new method that combines sequence-specific DNA extraction with microarray-based comparative hybridization to provide a robust and accurate picture of the transposon content of a given genome, and highlight the differences between individual genomes. Our work shows the high degree of transposon mobility among yeast strains and the potential for transposon mobility during outcrossing. We map full-length Ty1 and Ty2s as well as Ty3 LTRs throughout SK1, a common unsequenced lab yeast strain. Finally, we show that this mapping method can be extended to determining the location of modified bacterial transposons inserted into the yeast genome, as part of a transposon mutagenesis library.

## Results

### Description of General Method

**Figure 1:**
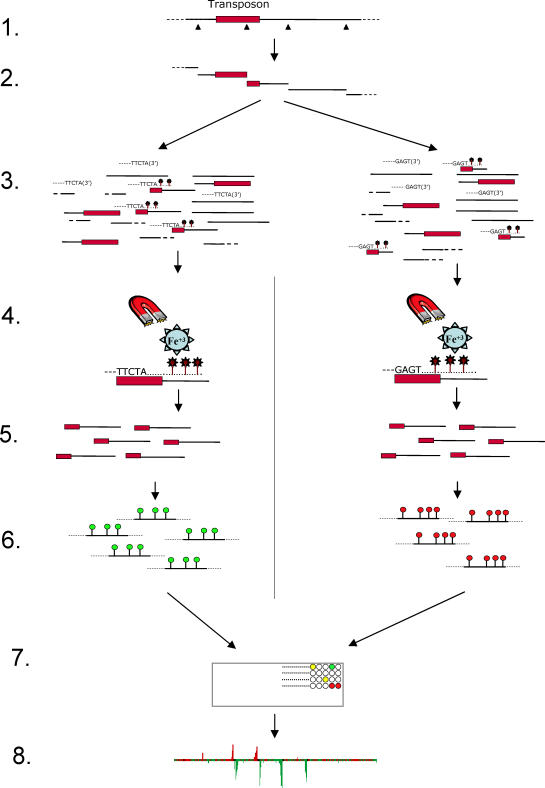
A General Schematic Diagram of the Steps Involved in Extracting, Labeling, and Identifying the Position of Transposons within a Genome Step 1. Genomic DNA is digested with a restriction endonuclease containing a cut site (triangle) within the transposon (red box). This results in multiple restriction fragments, including ones containing transposon and contiguous flanking DNA. Step 2. Digested DNA (which may be pooled from multiple separate digests) is mixed with oligonucleotide probes that have been designed to anneal to specific sequences within the transposon. Separate probe sets anneal to different transposons (as shown), or separate genomic DNA samples are used to compare transposon content from different sources. Step 3. After heat denaturation and reannealing, the mixture is incubated in the presence of a DNA polymerase and dNTPs, one of which is biotinylated (stick with star atop it). This allows specific extension from the annealed 3′ probe termini. Step 4. Extended probes and their annealed templates are purified away from the mixture using magnetic streptavidin-coated beads (star labeled with Fe^+3^). Step 5. The extracted templates are released by heating. Step 6. The templates are labeled using Cy3- or Cy5-labeled nucleotides (green and red lollipops, respectively) in the presence of random primers and a DNA polymerase. Step 7. Differentially labeled DNAs are hybridized to a microarray slide with features distributed throughout the genome. After washing, the array is scanned to identify features (circles) that are common to both DNA sources (yellow circles) or that have been differentially extracted (green or red circles). Step 8. The log_2_ ratio of signal intensity for the two dyes is quantitated and graphically represented along each chromosome to identify contiguous segments of differential signal that correspond to the DNA flanking the original transposons.

While individual members of a family of transposons are highly conserved, the flanking sequences into which different members insert are likely to be unique. Thus, transposon locations can be determined by identification of their contiguous DNA sequences. In order to isolate DNA fragments containing sequences that flank specific transposons, we digested whole genomic DNA with three different restriction endonucleases, pooled the digested DNA, and combined it with oligonucleotide probes designed to anneal to specific segments of selected transposons (Figure 1, steps 1–3). Incubation of the DNA and oligonucleotides in the presence of a DNA polymerase and nucleoside triphosphates, one of which is biotinylated, resulted in the addition of biotinylated bases to the extended primer. Subsequently, magnetic beads coated with streptavidin were added and used to separate the annealed fragments away from all other genomic DNA fragments (Figure 1, step 4). The extracted DNA fragments were released from the beads and fluorescently labeled using either Cy3- or Cy5-dUTP (or dCTP) in the presence of random primers and exo− Klenow fragment (Figure 1, steps 5 and 6), and then hybridized to dense whole genome oligonucleotide microarrays of the S. cerevisiae genome (Figure 1, steps 7 and 8). By analyzing comparative hybridization signals, we minimized noise from nonspecifically extracted fragments and accentuated differences in extracted fragments from two different sources of genomic DNA, or from extraction of the same genomic DNA using oligonucleotide probes specific to different families of transposons. For our experiments, we used Agilent yeast whole genome microarrays, because they consist of >40,000 oligonucleotide features, each 60 bases long, spaced approximately every 300 bases along the yeast genome. Tys and other repeat sequences were purposely avoided during the construction of this array.

### Comparison of Isogenic Strains

**Figure 2:**
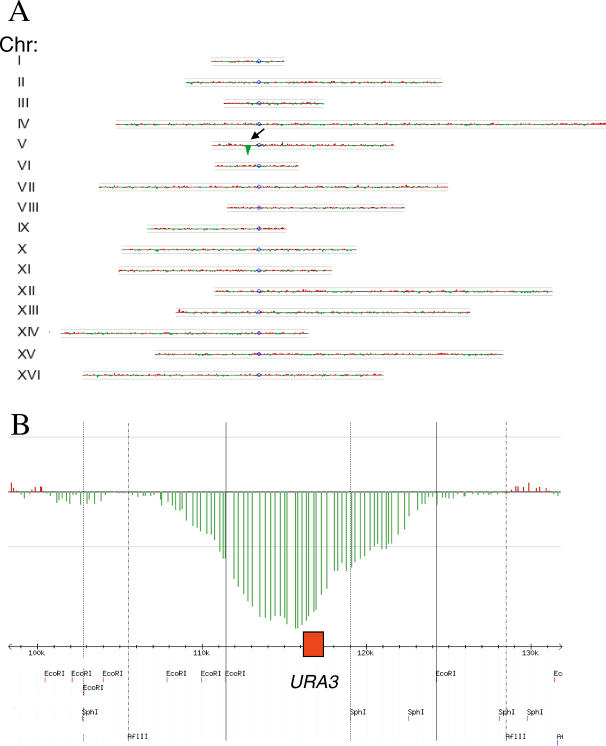
Identifying a Unique Ty1 Element in Otherwise Isogenic Strains (A) Two isogenic yeast strains (FY5 and FY2) differ only by the presence of a Ty1 insertion in Chromosome V within the *URA3* gene in FY2. After labeling transposon extracted DNA from FY2 with Cy3 (green) and transposon extracted DNA from FY5 with Cy5 (red), the labeled DNA was hybridized to an Agilent yeast whole genome microarray with >40,000 unique features (yeast repetitive DNA was avoided during array construction). Log_2_ ratio of hybridization for each feature along each chromosome is shown plotted in genome order using the TreeView Karyoscope function. The one region of significant differential hybridization is marked with an arrow. The grey horizontal lines above and below each chromosome correspond to 3-fold differential hybridization intensity. (B) Zoom view of a portion of Chromosome V and the peak of differential hybridization corresponding to the ∼8 kb surrounding *URA3* (red box). The positions of nearby restriction sites for the enzymes used initially to digest genomic DNA are shown based on a GBrowse view of the region from SGD.

We first used this method to identify one new Ty1 insertion in otherwise isogenic strains containing >40 Ty1 and Ty2 elements. FY2 and FY5 are isogenic derivatives of S288c, differing only by the presence of a full-length Ty1 element in the *URA3* gene of FY2 [28]. We annealed digested DNA from each strain with a set of probes corresponding to internal sequences common to both Ty1 and Ty2. Analysis of the log_2_ ratio of normalized intensity per feature showed near-perfect agreement between the two strains (i.e., minimal differential hybridization), except for a span of ∼8 kb on the left arm of Chromosome V centered on the *URA3* gene that showed 2- to 8-fold increased hybridization for FY2 (Figure 2A). Closer inspection of this peak (Figure 2B) showed that it correlated roughly to the summation of the cleavage sites flanking *URA3* for the three restriction endonucleases (AflII, EcoRI, and SphI) initially used to digest the two DNA samples. This result demonstrates that the extraction and mapping method can identify the location of a single differential transposon insertion.

### Comparison of Two Unrelated Strains

**Figure 3:**
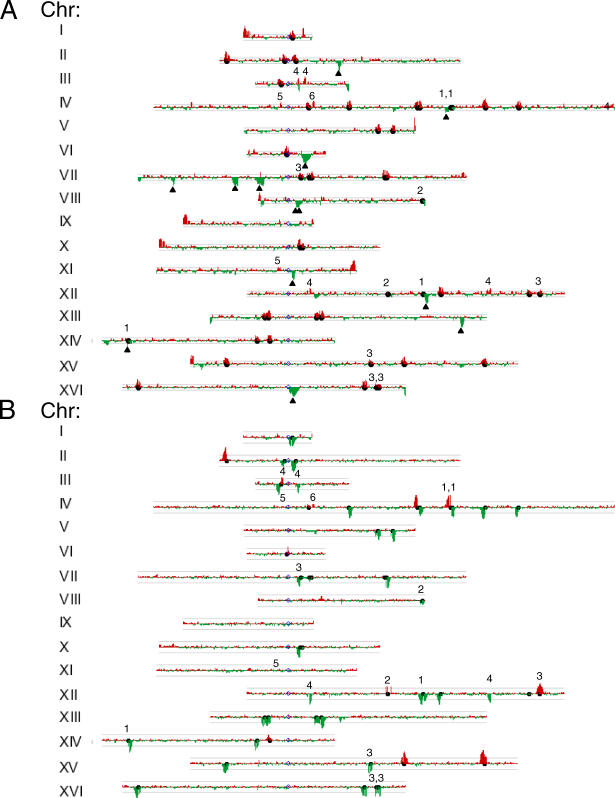
Validation of Whole Genome Transposon Analysis Using Two Sequenced Strains of S. cerevisiae (A) Whole genome comparison of full-length Ty1 and Ty2 elements from yeast strains RM11 and S288c after hybridization to the same Agilent yeast whole genome microarray. Black circles indicate the position of Ty1 or Ty2 full-length elements annotated for S288c in SGD. Triangles indicate full-length Ty2 elements identified in the sequence of RM11. Red peaks correspond to potential Ty1 or Ty2 elements present in S288c, while green peaks correspond to potential Ty1 or Ty2 peaks present in RM11. (B) Comparison of location of Ty1 full-length elements (green) and Ty2 full-length elements present in S288c. Symbols are as above. Numbers above various peaks refer to the following: 1, false-negative S288c elements obscured by overlapping elements in RM11; 2, false-negative S288c elements located in regions that are poorly represented by features on the array; 3, false-negative S288c elements that missed the criteria for calling a peak; 4, unannotated Ty1 full-length elements in S288c confirmed in this study; 5, false-positive peaks due to borderline elevated differential hybridization; 6, false-positive peaks corresponding to non-Ty repetitive elements in the genome. Grey horizontal lines above and below the central line for each chromosome correspond to a 3-fold difference in normalized ratio of Cy5 and Cy3 signal intensity.

We next validated the method by comparing the Ty1 and Ty2 content of two sequenced strains of S. cerevisiae, S288c and RM11. The S288c sequence was the first published eukaryotic genome [26], and the transposon content of this strain has been subjected to extensive analysis [3,29–31]. RM11 was isolated from a California vineyard, and was recently sequenced at the Broad Institute (http://www.broad.mit.edu/annotation/fgi). Analysis of the two sequences has shown that although they share many solo LTR elements they have no common full-length Ty1 or Ty2 elements (A.G., L. Kruglyak, and S. P., data not shown) and that RM11 has no full-length Ty1 elements at all (Table S1). We used the same set of probes to extract both Ty1- and Ty2-associated fragments from either S288c or RM11 restriction endonuclease–digested genomic DNA. We labeled the RM11 fragments with Cy3 (green) and the S288c fragments with Cy5 (red), and then hybridized the labeled DNA to an array. After washing and scanning, the relative hybridization intensity was calculated for each oligo feature on the array, and these values were aligned by position along each chromosome. We scanned the values on each chromosome and designated a location as a potential transposon peak if five or more consecutive features had log_2_ ratios of hybridization signal greater than 1.58, corresponding to a 3-fold difference in relative intensity of one dye over the other. Peaks located within 10 kb of one another were joined. These criteria were chosen to optimize the balance between false positives and false negatives. As shown in Table 1 and Figure 3A, we observed 48 peaks for S288c and 23 peaks for RM11. Changing the cutoff value or the number of consecutive probes meeting the cutoff increased either the false-positive rate or the false-negative rate (data not shown).

**Table 1:**
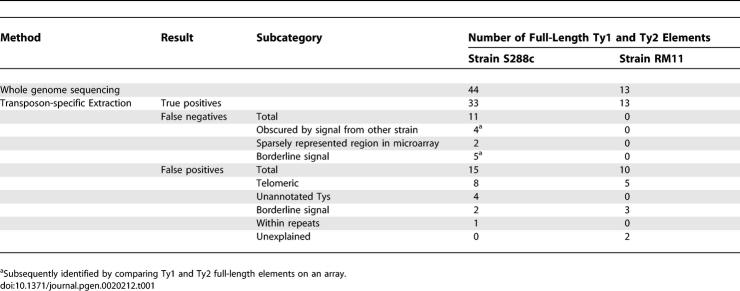
Comparison of Ty1 and Ty2 Full-Length Retrotransposons Identified by Whole Genome Sequencing Versus Transposon-Specific Extraction

Our peak-calling method identified the location of the vast majority of expected full-length Ty1 and Ty2 elements in each genome, based on prior whole genome sequencing of the two strains. For S288c, there are 44 annotated full-length Ty1 or Ty2 elements. Several of these occur in tandem duplications, and the arrays cannot easily distinguish a single Ty element from multiple tandem elements. We correlated the position of 33 full-length elements with observed S288c peaks, corresponding to detection of 75% of all Ty1 or Ty2 locations in a single experiment (red peaks with underlying black circles in Figure 3A). Among the 11 false negatives, four were obscured by overlapping peaks in RM11 (black circles with a “1” above them in Figure 3A), two were at a telomere or close to the rDNA locus, regions of the genome that are poorly represented by features on the array (black circles with a “2” above them in Figure 3A), and five missed the criteria for calling a peak (black circles with a “3” above them in Figure 3A). We carried out a second array experiment for S288c using probes that extract only full-length Ty1 elements (subsequently labeled with Cy3) or full-length Ty2 elements (subsequently labeled with Cy5). This second array demonstrated the presence of Ty1–Ty2 tandem elements (e.g., on Chromosome IV in Figure 3B), eliminated the fortuitous overlap between the S288c and RM11 peaks, (black circles with a “1” above them in Figure 3B), and accentuated the differential hybridization signal in all five borderline cases (black circles with a “3” above them in Figure 3B). The Ty1 versus Ty2 array resolved nine false negatives from the first array. However, in the two cases of sparse genomic representation by the oligo features on the array, the second experiment did not yield peaks that met our criteria (black circles with a “2” above them in Figure 3B).

For RM11, there are 13 predicted full-length Ty2 elements. We detected all 13 on the single array comparing RM11 with S288c, and the peaks were generally broader and more robust than for S288c (Table 1; Figure 3A). This is likely due to the greater number of features corresponding to RM11 insertions, since the number of features associated with the S288c insertions was purposefully minimized during the array design.

While the arrays identified real Ty1 and Ty2 elements, there were also a number of potential false-positive peaks. Eight of 15 false positives for the S288c genome were in telomeric or subtelomeric regions, and were likely a consequence of the tandem repetitive nature of these regions and the differences in X and Y inserts at different telomeres in different strains. These telomeric peaks were present in comparisons between the two strains but were much less apparent in comparison of the two different probe sets for the same strain (compare chromosome ends between Figure 3A and 3B). For other false positives, the number of features above the threshold level was marginal, but did meet our peak-calling criteria. These false positives did not correspond to peaks in the Ty1 versus Ty2 array (regions marked by a “5” or “6” in Figure 3A and 3B). These false positives could represent probe-specific cross hybridization or possibly differences in the nonspecific extraction and/or hybridization of genomic elements whose copy number varies between strains (e.g., peak for the *ENA* family region on Chromosome IV, marked by a “6” in Figure 3A). Control array experiments, comparing extraction and hybridization of S288c and RM11 in the absence of probes, show several peaks at telomeres and in repeats (data not shown).

Four peaks from DNA derived from S288c were unannotated in the *Saccharomyces* Genome Database (SGD) (http://www.yeastgenome.org) but also showed up on the Ty1 versus Ty2 array (regions with a “4” above them in Figure 3A and 3B). Two were present on the right arm of Chromosome III, centered at ∼145000 and ∼169000. The official map of S288c shows several solo LTRs at these locations but no full-length Ty1 or Ty2 elements. We confirmed by sequence analysis that these two unannotated peaks are in fact Ty1 elements, and their organization is complex (data not shown). In particular, two Ty1s are present at ∼169000, in a head-to-head orientation. Interestingly, the Tys on Chromosome III have been previously described and their polymorphic distribution in different yeast strains studied [16,32–34]. Their existence is discussed in the original report of the complete Chromosome III sequence [35]. Two other unexpected peaks were on Chromosome XII, one centered at ∼219000 and the other at ∼816000. The former is listed in SGD as an ORF, but is annotated as a partial Ty1 element. The latter has a solo LTR and a tRNA listed in SGD, but no apparent Ty elements. We used combinations of PCR primers on either side of the peak positions, as well as primers internal to Ty1 and Ty2, to confirm the presence of the predicted Ty element, which is inserted at base 818470, midway between the preexisting LTR and tRNA at this location (data not shown).

In summary, using two different arrays and three sets of extraction probes, our mapping technique identified the position of ∼95% of the true-positive full-length Ty1 or Ty2 elements in S288c and 100% of the expected Ty2 elements in RM11. By comparing results from the two arrays, we eliminated most false positives and identified several misannotated or unannotated transposons in the S288c genome.

### Extension of Technique to Unmapped Strains

**Figure 4:**
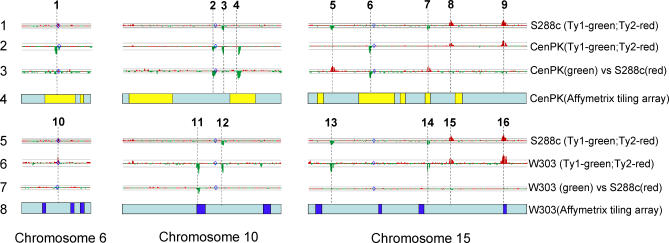
Comparison of Full-Length Ty1 and Ty2 Elements on Chromosomes VI, X, and XV in Strains S288c, CEN.PK, and W303 Rows 1, 2, 3, 5, 6, and 7 are based on transposon extraction data from Agilent yeast whole genome microarrays. Rows 4 and 8 correspond to Affymetrix tiling arrays probed with either CEN.PK DNA or W303 genomic DNA. For rows 1, 2, 5, and 6, digested genomic DNA as noted was extracted with either Ty1-specific or Ty2-specific sets of probes. For rows 3 and 7, digested genomic DNA was extracted with the set of common Ty1 and Ty2 probes. Numbers above vertical dashed lines refer to examples of transposon insertions of interest. Grey horizontal lines above and below the central line for each chromosome correspond to a 3-fold ratio of signal intensity. In rows 4 and 8, pale blue rectangles correspond to regions of CEN.PK and W303, respectively, derived from its S288c parent. In row 4, yellow rectangles correspond to regions of CEN.PK derived from its non-S288c parent. In row 8, dark blue rectangles correspond to regions of W303 derived from its non-S288c parent.

We next compared the pattern of transposons in S288c with those in two common lab strains, CEN.PK and W303. Both strains were originally derived from crosses between S288c and unrelated strains, although the detailed histories and origins are not completely documented. Previous work has shown that CEN.PK and W303 are chromosomal patchworks, with segments of S288c sequence interspersed with segments from the other parent [36,37]. We wanted to correlate the position of transposons in these hybrid strains with the strain origin of the DNA into which they were inserted. Therefore, we determined the transposon content of S288c, CEN.PK, and W303 (using Ty1- or Ty2-specific probes) and additionally compared S288c with CEN.PK or W303 using the set of probes that detected both Ty1 and Ty2 full-length elements Independently, we hybridized CEN.PK and W303 genomic DNA to Affymetrix yeast tiling arrays, which contains 25-mer oligonucleotide features based on the S288c sequence, and applied the SNPScanner algorithm to determine the location of segments derived from each parent along each chromosome [37] (examples in Figure 4, and whole chromosome data for W303 in Figure S1). We then overlaid the transposon information for CEN.PK and W303 with the whole genome SNPScanner predictions. As seen in Figure 4, in most cases the strain of origin of the CEN.PK segment could explain the presence or absence of a transposon at a given locus. Of 54 transposon peaks in S288c and/or CEN.PK, 24 corresponded to transposons observed in both CEN.PK and S288c, and these occurred in regions of the CEN.PK genome derived from S288c (Figure 4, examples 3, 8, and 9). Similarly, in 23 cases where transposon peaks were present only in S288c or in CEN.PK, the corresponding portion of the CEN.PK genome was not derived from S288c (Figure 4, examples 4, 6, and 7). However, several anomalous cases were also observed. In one case (Figure 4, example 1) both strains had a transposon peak in the same location, canceling out the signal in the CEN.PK versus S288c array, although this portion of CEN.PK was not of S288c origin. Analysis of the region in the two strains showed that these were independent events; a Ty2 is present in S288c and a Ty1 is present in CEN.PK, with the insertion sites offset from one another by 170 bases. In four cases, a Ty element was present in CEN.PK, but not in S288c, despite the expectation from the tiling array that the insertion was in an S288c-derived region (e.g., Figure 4, example 2). We have confirmed two of these new insertions in CEN.PK, including the one shown in Figure 4 on Chromosome X, as well as another on Chromosome XIII, at ∼504500. Conversely, there were two cases in which a Ty element was present in S288c but not present in CEN.PK, although the respective portion of the CEN.PK genome is derived from S288 (Figure 4, example 5, as well as another on Chromosome I). Sequencing of these two regions in CEN.PK revealed the absence of full-length Ty elements or even LTRs.

A similar pattern was seen with W303. Comparing S288c and W303, 54 transposon peaks were present. Of these, 44 could be explained based on the origin of the particular segment in W303 (Figure 4, examples 10–16). Four peaks were absent from W303, despite the segment appearing to be derived from S288c; three peaks were present only in W303, despite the segment appearing to be derived from S288c; and in three cases Ty peaks were present in similar locations in S288c and W303, although the corresponding W303 segment was not of S288c origin.

**Figure 5:**
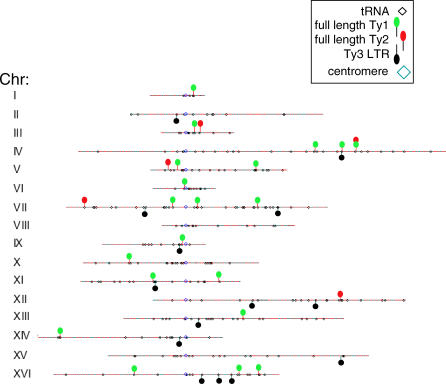
Transposon Map of SK1 The positions of Ty1 and Ty2 full-length elements and Ty3 LTR elements in strain SK1 are shown, based on Agilent yeast whole genome microarray (Ty1 and Ty2) and Agilent ORF array (Ty3 LTR) analysis of this uncharacterized genome.

We next examined the transposon content of SK1, a commonly studied laboratory strain unrelated to S288c. Using a variety of transposon-specific extraction probes we were able to identify 20 potential full-length Ty1 elements, five potential Ty2 elements, and 14 potential Ty3 LTRs. Based on these data we generated the transposon map for SK1 shown in Figure 5 (the approximate coordinates of the insertions are given in Table S2). In 94% of the predicted insertion sites, the peaks for the full-length element or LTR are closely linked to the known locations of tRNA genes, as expected from the known preferences of yeast retrotransposons. We confirmed our predicted placement for seven Ty3 LTRs and four unique Ty1/Ty2 full-length elements, using a combination of PCR and sequencing. Thus, our technique can quickly and accurately assign transposon locations in an otherwise unsequenced strain. In six cases the positions of transposons in S288c and SK1 overlapped one another. Detailed sequence analysis will be required to determine whether these are the same evolutionarily conserved elements or different elements inserted in similar locations.

### Mapping Transposons Used for Gene Tagging

**Figure 6:**
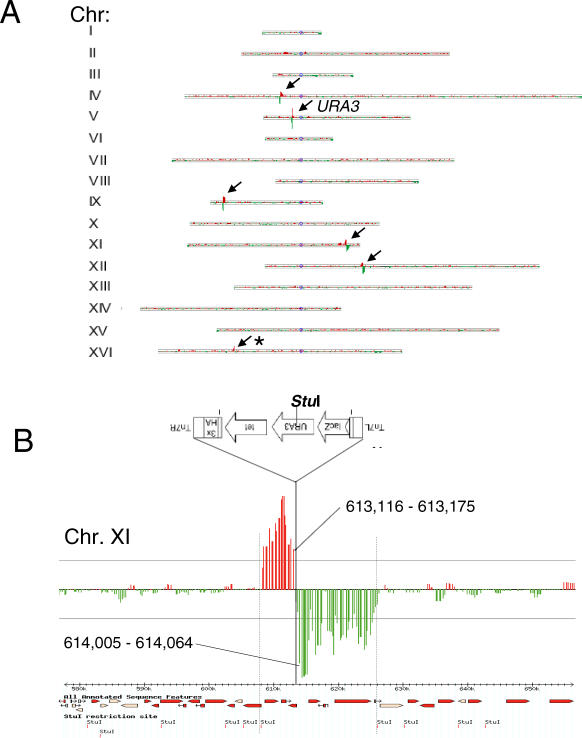
Analysis of Modified Bacterial (“Artificial”) Transposon Insertions in the S. cerevisiae Genome (A) Positions of five independent pooled artificial transposons from a yeast insertion library were determined after extracting StuI-digested yeast genomic DNA with probes designed to correspond to either strand at the 5′ or 3′ end of *URA3,* labeling with Cy3 and Cy5, respectively, and hybridizing to an Agilent yeast whole genome microarray. Arrows signify locations of significant differential hybridization. *“URA3”* indicates the actual *URA3* locus on Chromosome V. The asterisk indicates an insertion on Chromosome XVI in which only the flanking region 3′ to the transposon is detected. Vertical lines above and below the horizontal for each chromosome represent the log_2_ ratio of hybridization intensity for Cy5 versus Cy3 at each feature along the Agilent yeast whole genome microarray. For each insertion, the actual insertion site, determined by sequencing, and the position of the first significant flanking features are as follows: Chromosome IV, 368020, and 367656–367715 and 368589–368648; Chromosome IX, 55576, and 55291–55350 and 55808–55867; Chromosome XI, 613654, and 612706–612765 and 614005–614064; Chromosome XII, 387226, and 386346–386405 and 387248–387307; and Chromosome XVI, 296609, and 296350–296409 to 297592–297651. (B) An enlargement of the region detected on Chromosome XI, showing the structure of the artificial transposon, its unique StuI site, the bases covered by the oligonucleotides in the features on either side of the transition from significant differential Cy5 labeling to Cy3 labeling, and the position of the actual insertion. The map of the region from GBrowse of SGD shows the position of StuI restriction sites in the region. Grey horizontal lines above and below the central line for each chromosome correspond to a 3-fold ratio of signal intensity. Note that the transposon inserted in opposite orientation relative to the chromosome numbering, and is therefore flipped in the figure.

A number of methods have been described for genetic screening based on randomly inserting modified bacterial transposon sequences (referred to here as artificial transposons) into plasmid-based yeast genomic libraries and then transforming pools of the yeast DNA containing the artificial transposons into the yeast genome by recombination [38–41]. This results in libraries of yeast clones, in which each clone is marked by a different bacterial insertional event, which can then be selected phenotypically. To test our method for identifying the location of artificial transposon insertions in the yeast genome, we first sequenced the insertion junctions of five independent *URA3*-marked Tn7-based artificial transposons present in a plasmid-based yeast genomic library [40]. In this way we knew the precise insertion site for each artificial transposon. The yeast DNA segments from the five plasmids were transformed into yeast strain FY3, and cells that had acquired uracil prototrophy by homologous recombination of the segments were chosen. We then purified genomic DNA from the transformed strains, pooled the DNA, digested the pooled DNA with StuI, and extracted fragments using probes specific to either the 5′ end or the 3′ end of *URA3.* We chose StuI because it cuts only once in the artificial transposon, in the center of the *URA3* region. The extracted DNA samples were labeled with Cy3 (5′ flanking) or Cy5 (3′ flanking), and hybridized to an Agilent yeast whole genome microarray. As shown in Figure 6A, we observed six obvious regions of significant differential labeling (arrows), and these corresponded closely to the five sequenced insertion sites, as well as *URA3* itself, on Chromosome V. Thus, our method can simultaneously identify multiple transposon insertions, each present in only a fraction of the population.

The resolution of our method for identifying artificial transposon insertion sites depends on both the density of the features on the array, as well as the location of flanking restriction sites. For example, as shown in Figure 6B, significant differential labeling was present on Chromosome XI, with a transition from significant differential Cy5 hybridization to significant differential Cy3 hybridization occurring between bases 612765 and 614005. Sequence data showed that the actual insertion site is at 613654, indicating a resolution of <1 kb. As seen in Figure 6B, the borders of the differential hybridization signals correspond to the nearest StuI sites surrounding the insertion site. By applying this criterion, we were able to accurately predict four of the five insertion sites (see legend for Figure 6A.) In the case of the insertion site on Chromosome XVI (Figure 6A, asterisk), the nearest flanking StuI site upstream of the insertion occurs before the next oligonucleotide feature on the array. Consequently, the Cy3 peak corresponding to upstream flanking DNA is missing. This limitation on the method could be addressed by pooling multiple restriction digests for extraction, as we did with the Ty analysis, or by using arrays with denser genome coverage.

## Discussion

Here we show that transposon-specific extraction combined with microarrays provides an accurate and efficient approach to identifying the location of polymorphic transposable elements throughout the yeast genome. By combining the power of comparative hybridization to identify differences between two samples, with a robust technique for sequence-specific DNA capture and purification, we are able to compare the transposon content of different strains, distinguish closely related Ty1 and Ty2 elements from the same strain, map the location of transposons in unknown strains, and identify artificial transposons inserted into yeast strains as mutagens or genetic markers. Differences in Ty position and content in strains other than the sequenced reference S288c have been reported anecdotally, and the presence of unannotated Ty elements associated with unusual, strain-specific genetic events is a frequently confusing finding. In the field of yeast genetics and genomics this technique has many immediate applications: comparing phenotypic differences between yeast strains, studying the evolutionary dynamics of transposons within the yeast genome, identifying and monitoring industrial and vineyard strains, and identifying potential sources of mutation associated with changes in the properties of yeast strains. A similar technique employing vectorette PCR has recently been applied to characterize strains carrying high copy numbers of Ty1 [42].

The power of the technique described here comes from its ability to examine the whole genome simultaneously and provide positional information for further analysis. Mapping S288c versus RM11 as a proof of principle, 75% of the known S288c Ty1 and Ty2 elements and 100% of the known RM11 Ty2 elements were correctly assigned based on a single array with a set of five Ty1/2-specific probes (Figure 3A). By using additional probe sets, comparing Ty1 to Ty2 within S288c (Figure 3B), we could correctly identify >95% of the expected Ty1 and Ty2 elements. Further, the consistent signal at several unexpected locations, using either probe set, directed us to the presence of four unannotated Ty1 elements, which we subsequently verified by PCR and sequencing. Other false negatives are likely due to sparse positional representation in particular regions of the array, and/or fortuitous restriction sites near the end of the transposon. These limitations may be ameliorated by denser oligonucleotide arrays, and by using controlled-shear genomic DNA instead of restriction endonuclease–digested DNA.

Beyond simple mapping of different strains, our combination of transposon mapping and comparison of nucleotide variation for CEN.PK and W303 using the Affymetrix tiling arrays (Figure 4) demonstrates a new way of examining hybrid genomes. By correlating transposons in the parent strain S288c, transposons in the progeny strains, and the parental origin of segments along the progeny chromosomes resulting from meiotic recombination, it is apparent that while most transposons in the progeny strains were inherited in the predicted manner, others may have arisen in the course of mating and/or meiosis, while still others appear to have been lost. These findings suggest the possibility that mating and/or outcrossing stimulates mobilization and gene conversion of transposons in yeast, and we are observing the consequences of these processes. Alternatively, the anomalous Ty positions in CEN.PK and W303 could be due to differences in transposon location in the specific S288c parent strain that was used in the initial cross from which these hybrid strains were derived. To distinguish from amongst these possibilities we are currently examining transposon location changes in controlled crosses.

Previous reports have used microarrays to identify the position of multiple artificial transposons inserted into prokaryotic genomes [43–47], as well as in *Arabidopsis* [48]. Our demonstration of identifying artificial transposon insertions into the yeast genome indicates that similar approaches can be applied to *S. cerevisiae,* where the results can be complemented by the wealth of available genetic and genomic tools. The method described here differs from previous approaches in not requiring ligation or PCR amplification, making it simpler, more robust, and freer from amplification bias. As in bacterial systems, the loci of multiple independent transposon insertion events that result in a given phenotype can be rapidly and simultaneously determined. Similarly, dense libraries of artificial transposon insertions into the yeast genome could be analyzed by our method to identify potential essential regions (i.e., chromosomal segments lacking insertions).

Since the extraction method we employ is completely generic, our approach can theoretically be applied to the examination of transposons or other variable genomic segments in any species for which microarrays are available. We have, however, considered potential constraints to the general use of our current approach. For example, a limitation is imposed by the density and genome coverage of the features on the array. This problem increases with genome size and sets a limit on the resolution of the results. The extraction techniques we have used can be modified to optimize capture of DNA segments >50 kb (J.D. and M.K., data not shown), which could then span multiple sparse flanking features. A lower-resolution alternative, especially if presence or absence of a transposon in a region is more important than precise mapping, could be the use of arrays made from overlapping bacterial artificial chromosomes that span an entire genome (e.g., [49]). In this case, masking of repetitive sequences by prehybridization of the array with *Cot-1* DNA would be essential. At the other extreme, comparative hybridization to two color full-genome tiling arrays, with complete coverage of a genome sequence, could theoretically provide close-to-precise position data if noise from repetitive sequences could be sufficiently masked or filtered from the analysis. Another limitation to the technique is identifying transposon differences between samples if differences occur within tandem arrays of transposons or if transposons insert into preexisting transposons and other repetitive sequences. This could be particularly problematic in mapping heterochromatic regions such as the *Drosophila* chromocenter [50]. Careful analysis of results from multiple probe sets on the same genomic samples, including ones that compare 5′ ends to 3′ ends of transposons, could help to distinguish differences in complex clusters of transposons by the orientation of insertions.

## Materials and Methods

### Strains and DNA.

All strains used were obtained from the Botstein lab collection, and included FY2, FY3, and FY5 (all derivatives of S288c); RM11-1a [51,52]; CEN.PK [53]; W303 [54,55]; and SK1 [56,57]. Genomic DNA was obtained by growing up 100-ml cultures in yeast peptone dextrose medium [58] and then purifying DNA using the Genomic DNA Buffer Set (Qiagen, http://www1.qiagen.com) and Genomic-tip 500/G (Qiagen). Purified DNA was stored frozen in water. Two to three micrograms of DNA were digested with AflII, EcoRI, or SphI (New England Biolabs, http://www.neb.com) as per manufacturer's instructions, then precipitated and resuspended in double-distilled water (all experiments except for bacterial transposon analysis in Figure 6, in which case only StuI-digested DNA was used). Equal volumes of differently digested DNA were pooled for subsequent extraction.

### Transposon-specific extraction.

Pooled restriction-digested DNA (500–2,000 ng) was mixed with various sets of oligonucleotide primers (referred to as “probes” and supplied by Qiagen) in a buffer containing dNTPs, one of which has an attached biotin group, and with HaploPrep Hybridization Buffer (Qiagen), which contains a thermostable DNA polymerase. The DNA and probe mixture was heat-denatured for 15 min at 95 °C, then transferred to a BioRobot EZ1 (Qiagen) and allowed to renature and extend for 20 min at 65 °C. Streptavidin-coated magnetic beads were then added to the mixture to capture the DNA attached to the biotinylated probes. After four high-stringency wash steps, the bound DNA was released from the beads by heating to 80 °C in Qiagen EB buffer. The supernatant was collected for fluorescent labeling. All capture and purification reagents were contained within the Qiagen HaploPrep Cartridge-48. Application of a closely related method, referred to as haplotype-specific extraction, has been previously described [59].

Probe design was based on the specific goal of the experiment. To capture DNA flanking both full-length Ty1 and Ty2 elements (Figures 2, 3A, 4, and 5), an alignment of all full-length Ty1 and Ty2 elements in the S288c genome was used to identify regions of maximal base conservation. To capture DNA flanking only full-length Ty1 elements or only full-length Ty2 elements (Figures 3B, 4, and 5), the same alignment was used to identify regions of maximal difference between Ty1 and Ty2. To capture DNA flanking the LTRs of Ty3 (Figure 5), an alignment of ten solo LTRs from S288c was used. For *URA3*-specific probes, probes corresponding to the 5′ and 3′ ends of the *URA3* coding region were synthesized. Positions of probes along their respective transposons and their orientations are shown in Table S3, and their sequences are available upon request.

### Microarray procedures.

Aliquots of DNA recovered by the transposon-specific extraction procedure were combined with random primers and labeled using Cy3- or Cy5-coupled dUTP or dCTP according to instructions in the BioPrime Array CGH Genomic Labeling Module (Invitrogen, http://www.invitrogen.com). This kit uses the exo− Klenow fragment of Escherichia coli DNA polymerase to extend from the random primers and add a fluorescently labeled nucleotide. The products of the polymerization reaction were purified through DNA Clean and Concentrator-5 spin columns (Zymo Research, http://zymoresearch.com), resuspended in double-distilled water, and the quantity and incorporation of dye were measured using an ND-1000 Spectrophotometer (Nanodrop, http://www.nanodrop.com). Comparative hybridization was then performed using Yeast Whole Genome 44K ChIP-on-chip Microarrays (slide number 1, Design ID number 012713) (Agilent Technologies, http://www.home.agilent.com), which consist of 44,290 60-mer features spaced at an average of 290-bp resolution throughout the yeast genome in varying strand orientation. Repetitive sequences, including full-length Ty elements and LTRs, are not represented on the array. Labeled extracted samples (500 ng of each) were combined, mixed with Agilent 10X control targets, heated to 95 °C, and then mixed with Agilent 2X hybridization buffer before being added to the microarray slide. Hybridization was carried out at 60 °C for 17 h. Slides were washed according to the Agilent SSPE Wash protocol, dried in acetonitrile, and then scanned using an Agilent Microarray Scanner. Data extraction, including dye normalization and spatial detrending, was done using the default settings of the Agilent Feature Extractor. The resulting log_2_ ratio of Cy3 and Cy5 signal in each feature was used to determine the location of sequences flanking transposons. Data from the arrays were graphically displayed using Java TreeView [60]. For one dataset (SK1 Ty3 LTR determination in Figure 5), the Agilent Yeast V2 Oligo Microarray was used. This array consists of 10,267 60-mer features representing 6,256 known ORFs in the yeast genome. All hybridization and wash conditions were as above except that 250 ng of each labeled extracted sample was used. Affymetrix (http://www.affymetrix.com) yeast tiling arrays were used to analyze the nucleotide-level differences between yeast strains (data for Figure 4) using published methods [37]. All microarray data are available in the Gene Expression Omnibus (accession number listed under Supporting Information) and as supplemental information at http://rd.plos.org/ _01 or at 10.1371/journal.pgen.0020212_02.

### PCR and sequencing procedures.

PCR primers were designed using Primer3 (http://frodo.wi.mit.edu/cgi-bin/primer3/primer3_www.cgi). PCR products were obtained by standard means using Taq polymerase (Roche, https://www.roche-applied-science.com). Products were purified through Zymo columns and sequenced by Genewiz (http://www.genewiz.com) using one of the PCR primers as the sequencing primer. PCR primer sequences are available upon request.

## Supporting Information

Figure S1 Affymetrix Yeast Tiling Array Data for Each Chromosome after Hybridization with W303 DNA Signals above the central horizontal line of each chromosome correspond to sequence polymorphisms relative to the S288c sequence. Contiguous regions with many polymorphisms are likely derived from a parent that is not S288c, while regions with very few polymorphisms are likely derived from S288c.
(113 KB PDF)

Table S1 Insertion Sites of Ty2 Elements in RM11 Relative to the S288c Sequence (30 KB PDF)

Table S2 Mapped Locations of Ty1 and Ty2 Full-Length Elements and Ty3 LTR Elements in Strain SK1 (23 KB PDF)

Table S3 Position of Oligonucleotide Probes Used in This Study (31 KB DOC)

### Accession Numbers

The Gene Expression Omnibus (http://www.ncbi.nlm.nih.gov/geo) accession number for the microarray data discussed in this paper is GSE6278.

## Abbreviations

LTR: long terminal repeat
ORF: open reading frame
SGD: *Saccharomyces* Genome Database
