# Supplementary material for: Global Mapping of Transposon Location
Source: PLoS Genet. 2006 Dec 15;2(12):e212. doi: 10.1371/journal.pgen.0020212 (PMC1698948; doi:10.1371/journal.pgen.0020212)

Supplementary Figure 1 for Gabriel et al, 2006

Data from Affymetrix Yeast tiling array hybridized with W303 DNA, for each chromosome. Signals above the central horizontal line of each chromosome correspond to sequence polymorphisms relative to the S288c sequence. Contiguous regions with many polymorphisms are likely derived from a parent that is not S288c, while regions with very few polymorphisms are likely derived from S288c.

Chromosome 1

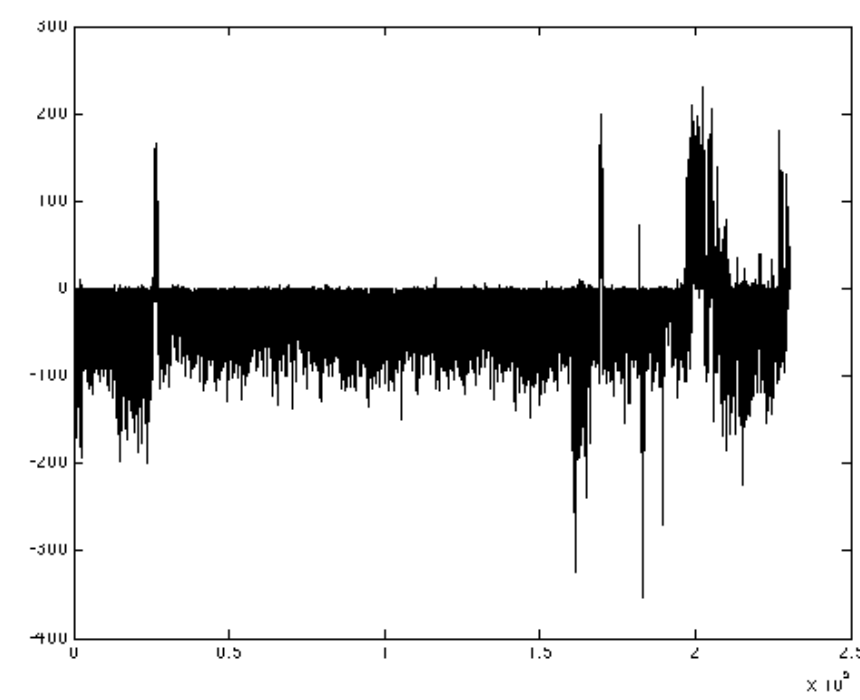

Chromosome 2

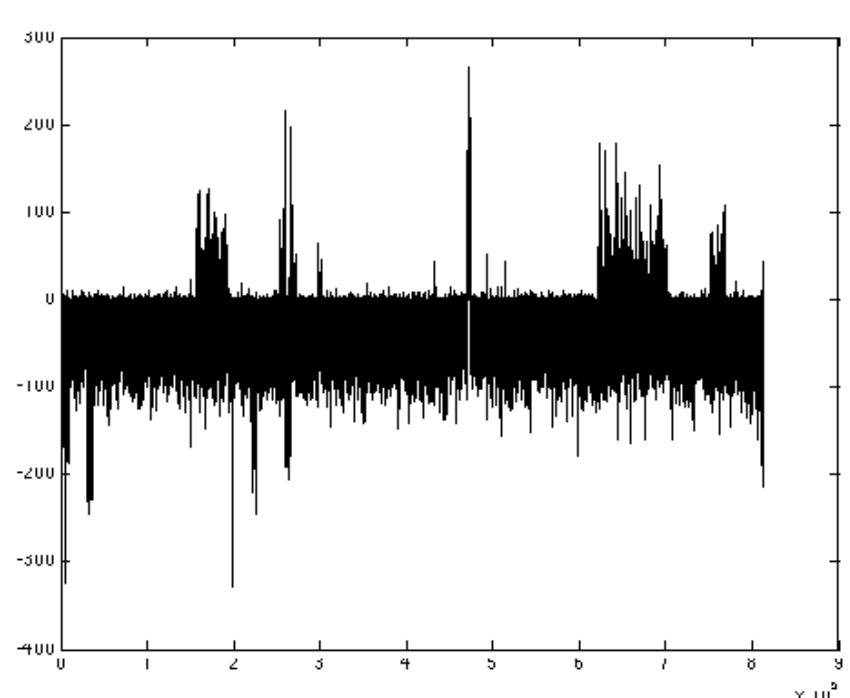

Chromosome 3

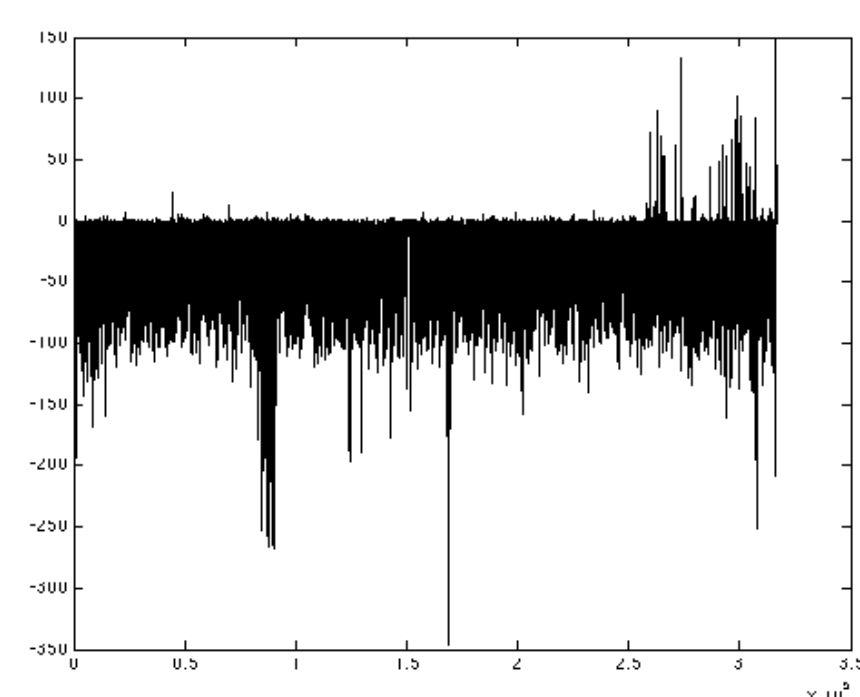

Chromosome 4

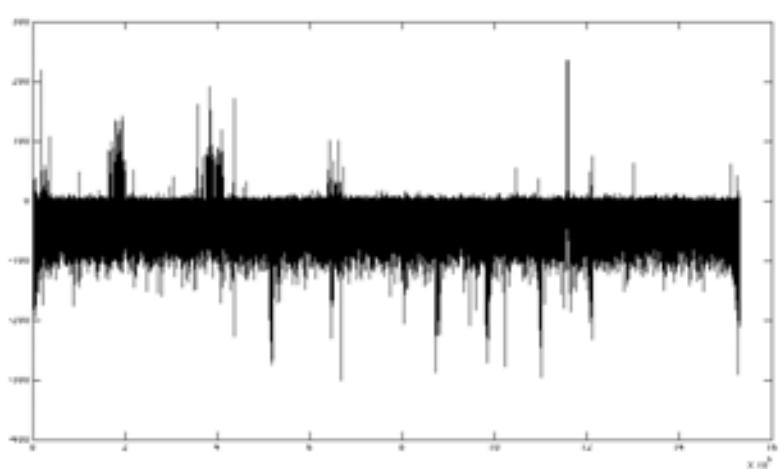

Chromosome 5

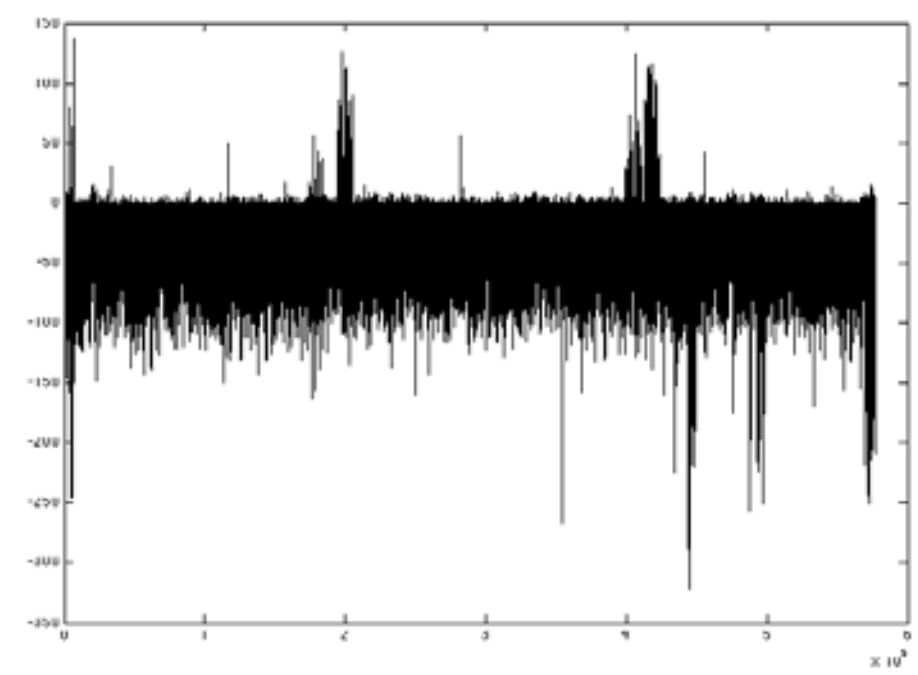

Chromosome 6

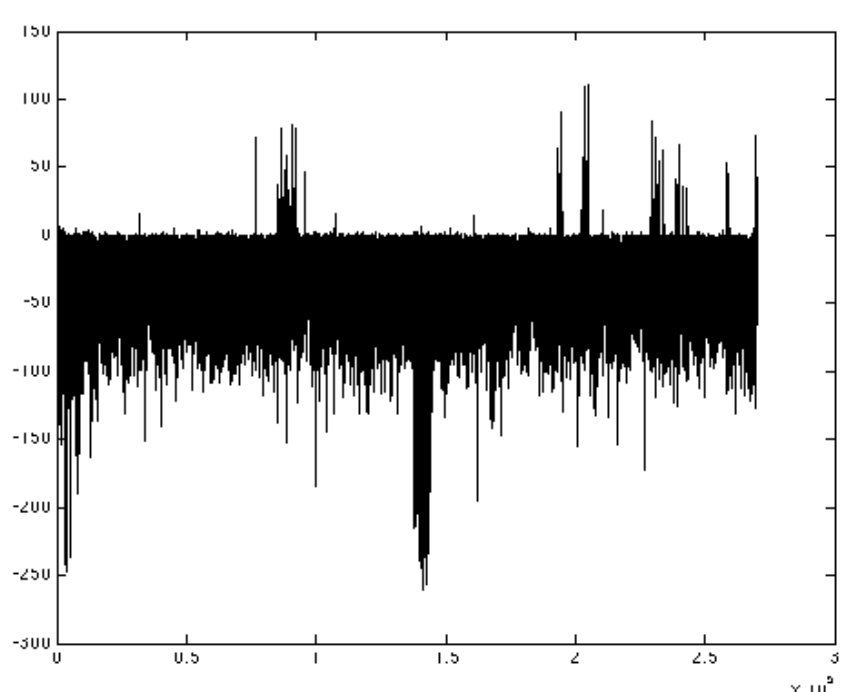

Chromosome 7

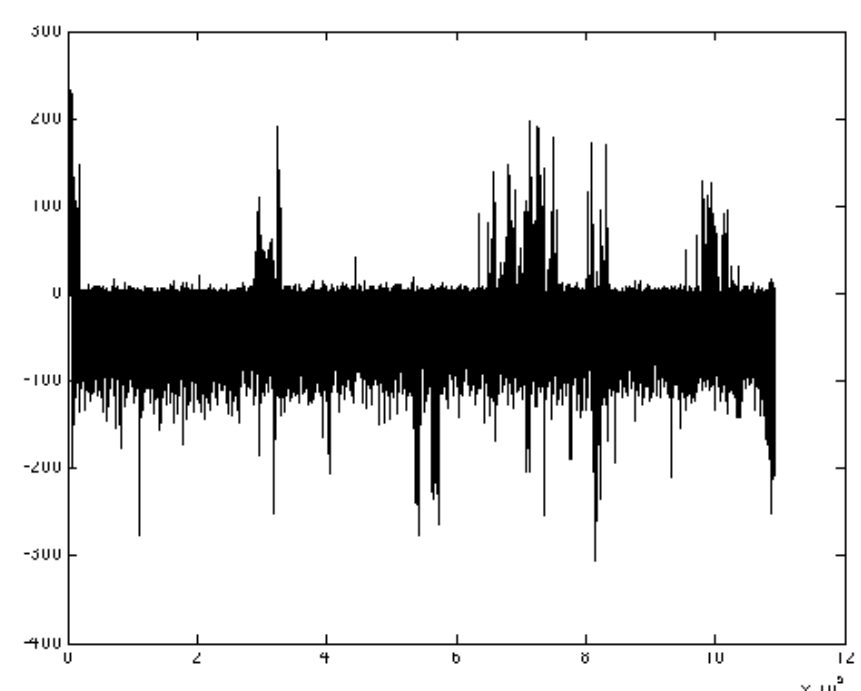

Chromosome 8

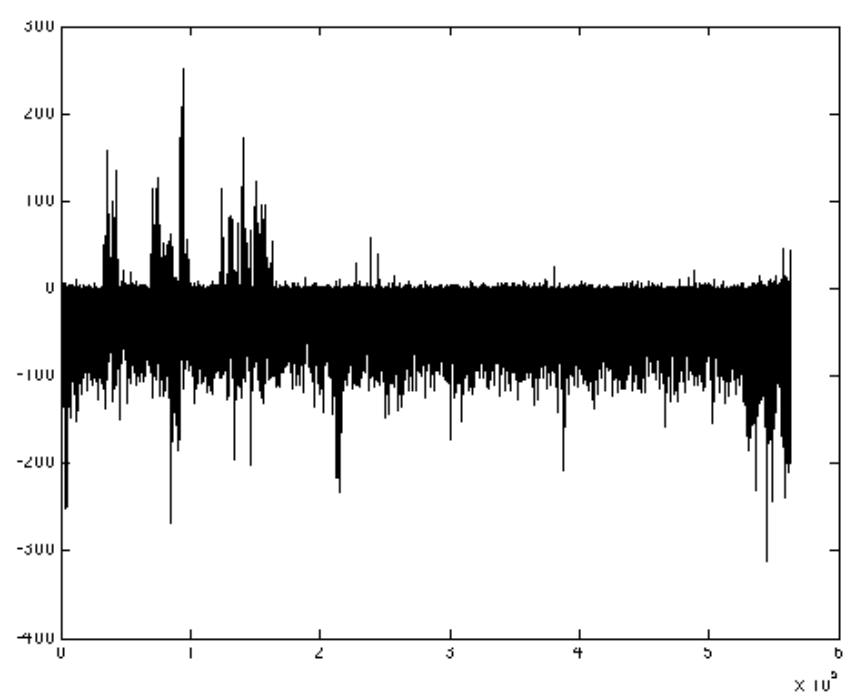

Chromosome 9

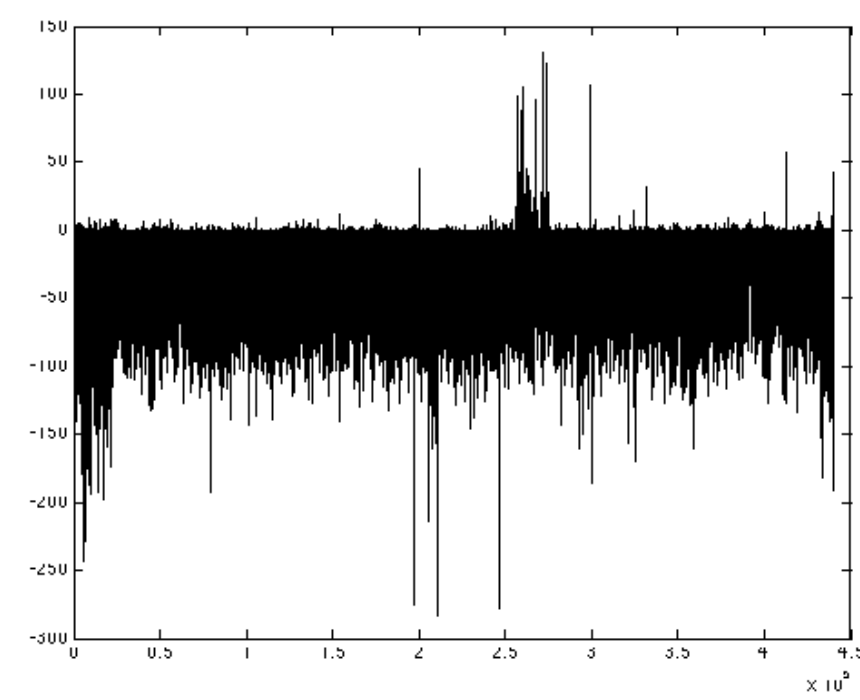

Chromosome 10

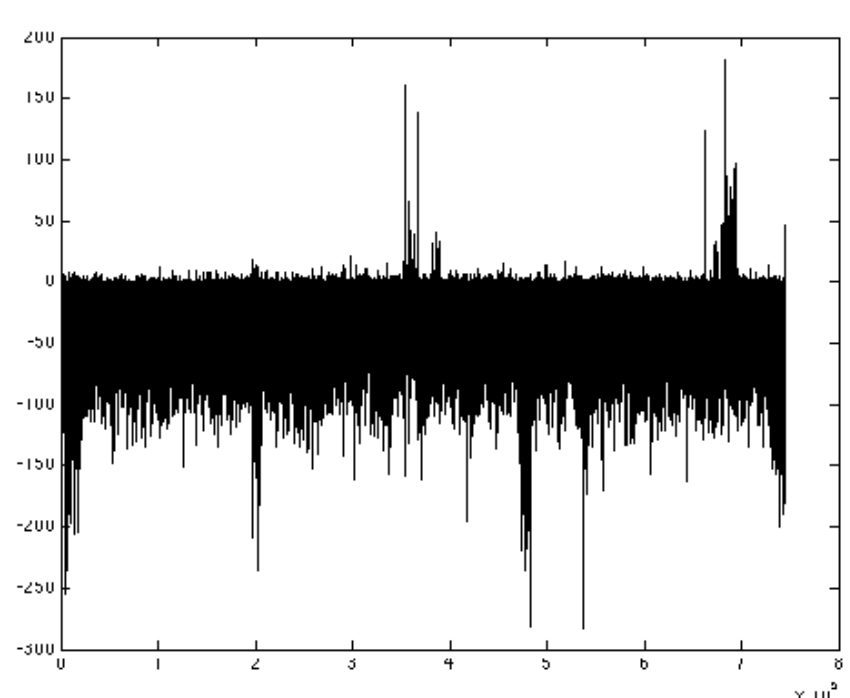

Chromosome 11

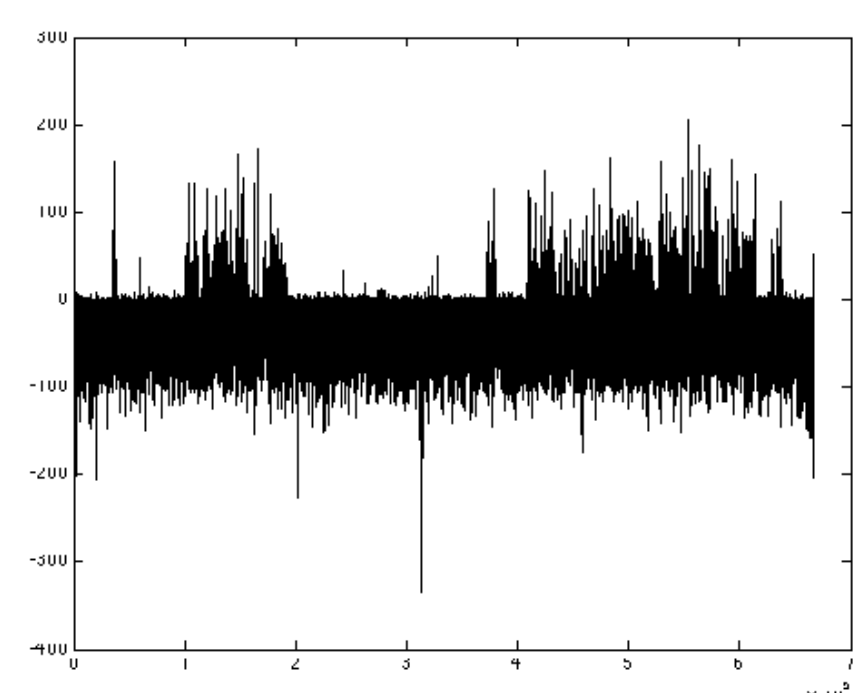

Chromosome 12

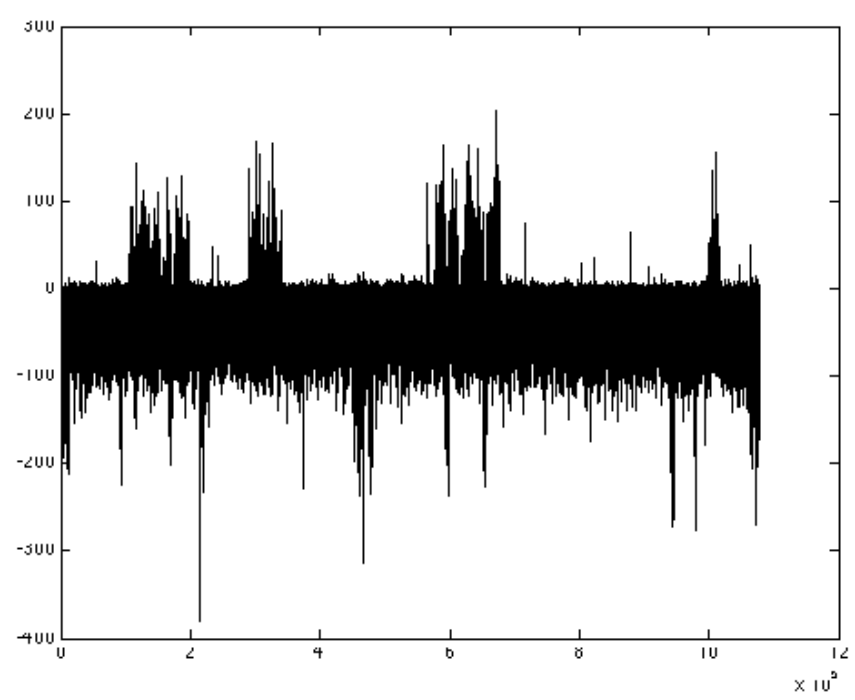

Chromosome 13

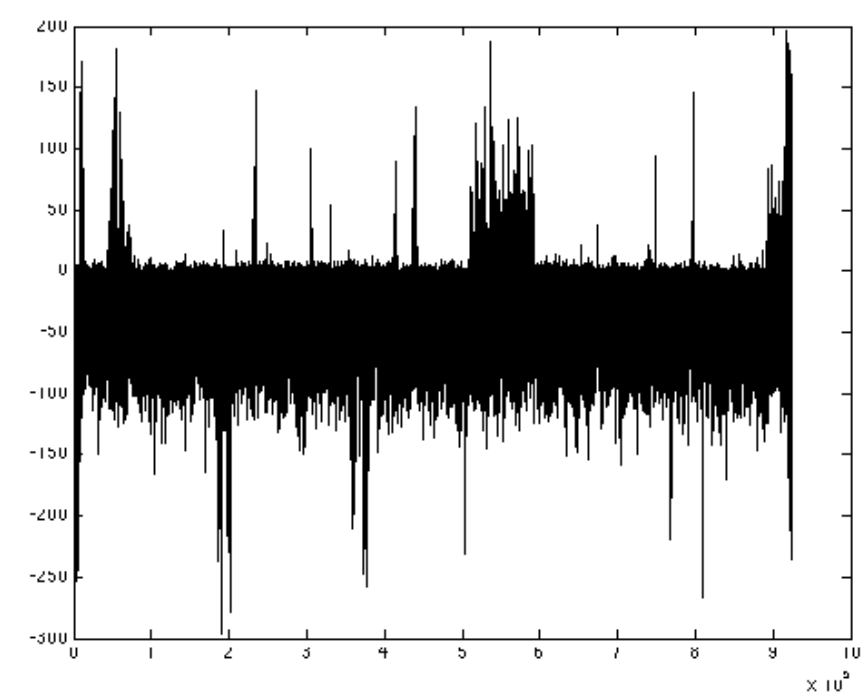

Chromosome 14

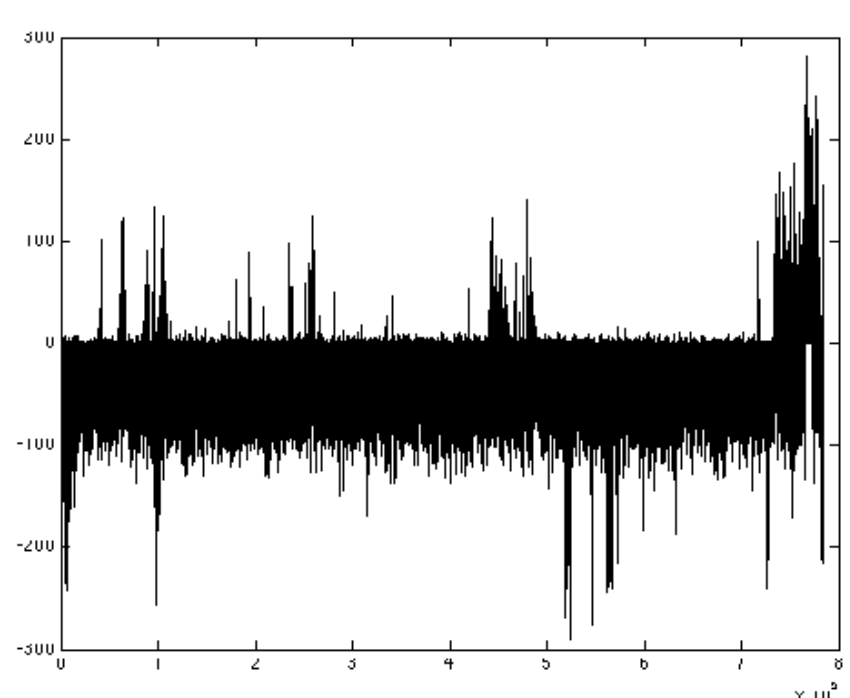

Chromosome 15

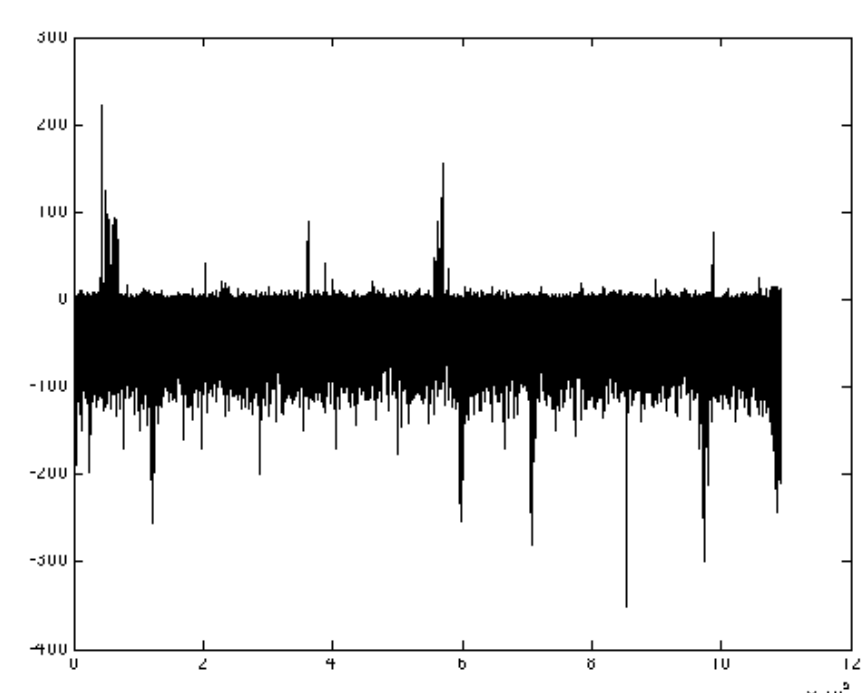

Chromosome 16

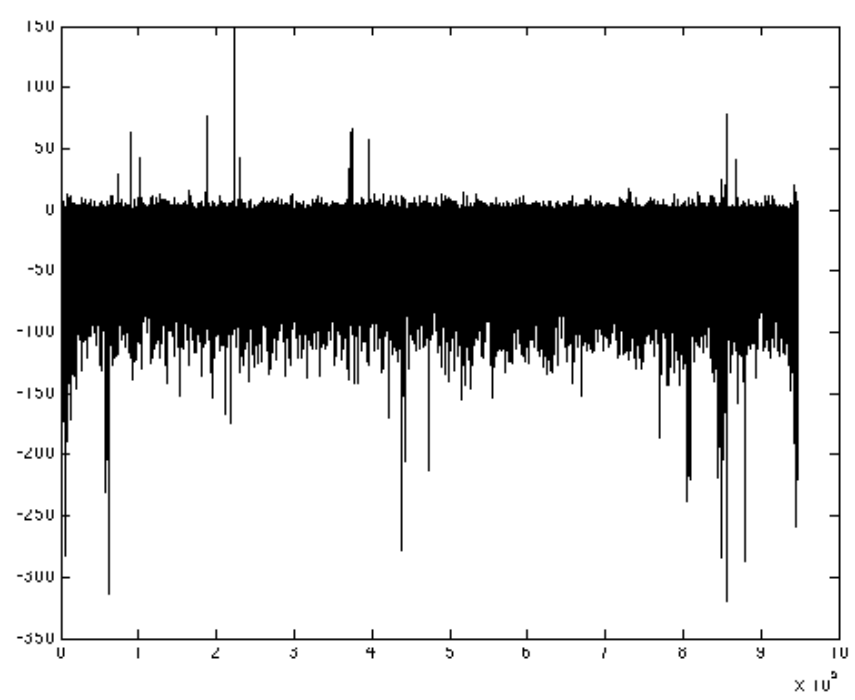

Supplement: Supplementary file 1 [file pgen.0020212.sg001.pdf]
