# Supplementary material for: Global Mapping of Transposon Location
Source: PLoS Genet. 2006 Dec 15;2(12):e212. doi: 10.1371/journal.pgen.0020212 (PMC1698948; doi:10.1371/journal.pgen.0020212)
Supplement: Supplementary file 2 [file pgen.0020212.st001.pdf]

Supplementary Table 1:

Insertion sites of Ty2 elements in RM11 and their insertion points relative to the S288c sequence, based on blast search analysis of the RM11 genome sequence.

Chromosome	Insertion point in S288c sequence
2	405558
4	991143
6	206608
7	115290
7	319065
7	402223
8	133572
8	Tandem with above
11	457478
12	605670
13	837678
14	96494
16	572154
