# Supplementary material for: Global Mapping of Transposon Location
Source: PLoS Genet. 2006 Dec 15;2(12):e212. doi: 10.1371/journal.pgen.0020212 (PMC1698948; doi:10.1371/journal.pgen.0020212)
Supplement: Supplementary file 3 [file pgen.0020212.st002.pdf]

Supplemental Table 2:

Mapped locations of Ty1 and Ty2 full length elements in SK1, giving range of location where peak relative hybridization signal is >3 x above the background.

Mapped location of Ty3 LTR elements in SK1, giving the position of the nearest tRNA gene, since Ty3 LTRs are known to insert within several bases of the start of a tRNA gene.

Ty1 and Ty2 coordinates for SK1	Range of peak signal (>3X signal increase)	
Chr1	181332-182402	Ty1
Chr3	148931-151406	Ty1
	173312-176979	Ty2
Chr4	973204-994087	Ty1
	1088958-1102355	Ty1
	1147971-1151040	Ty1
	1156272-1163036	Ty2
Chr5	77856-82063	Ty2
	106484-117635	Ty1
	438618-449769	Ty1
Chr6	131331-147106	Ty1
Chr7	73124-78114	Ty2
	433577-447581	Ty1
	543447-548141	Ty1
	785362-799622	Ty1
Chr9	337006-338425	Ty1
Chr10	193006-206623	Ty1
Chr11	296734-304239	Ty1
	572572-581018	Ty1
Chr12	788241-797465	Ty2
Chr13	502742-512783	Ty1
Chr14	95896-106996	Ty1
Chr16	334307-343365	Ty1
	770694-782032	Ty1
	843777-859724	Ty1
Ty3 LTR	Position of nearest tRNA	
Chr2	197497-197570	
Chr4	1095456-1095365	
Chr7	328586-328657	
	876399-876471, 878820-878715	
Chr9	325746-325818	
Chr11	307862-307789, 313117-313046	
Chr12	427133 -427204	
	687934 -687861	
Chr13	321218 -321146	
Chr14	602311 -602384	

Chr15	976416 -976488	
Chr16	622628 -622537	
	689562 -689643	
	744282 -744353	
