# Supplementary material for: Global Mapping of Transposon Location
Source: PLoS Genet. 2006 Dec 15;2(12):e212. doi: 10.1371/journal.pgen.0020212 (PMC1698948; doi:10.1371/journal.pgen.0020212)
Supplement: Supplementary file 4 [file pgen.0020212.st003.doc]

Supplementary Table 3: Position of oligonucleotide probes used in this study

| Targeted element | Probe Name^1^ | 5’ base | 3’ base | Reference Sequence |
| --- | --- | --- | --- | --- |
| Ty1-specific |  |  |  |  |
|  | 743F | 697 | 713 | Ty1^2^ |
|  | 793R | 761 | 740 | Ty1 |
|  | 3238F | 3183 | 3196 | Ty1 |
|  | 3188R | 3184 | 3167 | Ty1 |
| Ty2-specific |  |  |  |  |
|  | 779F | 4187 | 4204 | Ty2^3^ |
|  | 729R | 4131 | 4118 | Ty2 |
|  | 3161F | 6570 | 6585 | Ty2 |
|  | 3197R | 6619 | 6601 | Ty2 |
| Ty3 LTR |  |  |  |  |
|  | 182R | 177 | 164 | Ty3^4^ |
|  | 279F | 260 | 275 | Ty3 |
| Common to Ty1 and Ty2 |  |  |  |  |
|  | 460-1R | 394 | 380 | Ty1 |
|  | 5100-1R | 5000 | 4981 | Ty1 |
|  | 391-1F | 347 | 364 | Ty1 |
|  | 491-2F | 476 | 490 | Ty1 |
|  | 5046-2F | 4961 | 4977 | Ty1 |
| URA3 |  |  |  |  |
|  | 5’URA3F | 231 | 252 | *URA3*^5^ |
|  | 5’URA3R | 432 | 410 | *URA3* |
|  | 3’URA3F | 788 | 803 | *URA3* |
|  | 3’URA3R | 969 | 952 | *URA3* |

^1^F and R refer to + or – orientation of the probe

^2^Ty1 sequence is Ty1-H3, Genbank accession number M18706.

^3^Ty2 sequence is Ty1-917, Genbank accession number X03840.1.

^4^Ty3 sequence is Genbank accession number M23367.1.

^5^*URA3* sequence is Genbank accession number K02207.
